# Microfluidic Concentration Manipulation via Controllable AC Electroosmotic Flow

**DOI:** 10.3390/mi16111288

**Published:** 2025-11-15

**Authors:** Jingliang Lv, Yulong Pei, Jianqi Sun

**Affiliations:** 1 College of Mechanical and Electrical Engineering, Northeast Forestry University, Harbin 150042, China; lvjingliang4174@nefu.edu.cn; 2 College of Civil and Transportation Engineering, Northeast Forestry University, Harbin 150042, China; 2023111866@nefu.edu.cn

**Keywords:** microfluidics, AC electroosmosis, concentration manipulation

## Abstract

The ability to precisely prepare microfluids with targeted concentrations is critical for numerous applications, including protein crystallization and drug efficacy evaluation. This study presents an efficient microfluidic method for the continuous preparation of fluids at desired concentrations utilizing AC electroosmosis (ACEO). Two miscible fluids of different initial concentrations are introduced through separate inlets. Target concentrations are achieved through ACEO-driven mixing, where fluid manipulation via electric signal and flow velocity control enables precise concentration adjustment at the outlet. To elucidate the concentration control mechanism via ACEO, we develop a three-dimensional numerical model coupling electric, flow, and concentration fields. Our results demonstrate that concentration modulation is significantly influenced by intrinsic fluid properties and external control parameters, including fluid viscosity, conductivity, axial fluid velocity, driving voltage, and signal frequency. Specifically, higher fluid viscosity and conductivity dampen electroosmotic flow, necessitating increased voltage to achieve target concentration. Axial fluid velocity determines the residence time in the mixing zone, directly affecting mixing efficiency and concentration control effect. The intensity of ACEO flow increases with applied voltage, enabling tunable mixing performance and outlet concentration. Overall, the simplicity of device design combined with precise concentration manipulation makes this method particularly attractive for applications requiring accurate fluid preparation.

## 1. Introduction

The advancements in microfluidic technologies over the past twenty years have positioned them as powerful tools for applications ranging from particle synthesis to pharmaceutical screening, owing to their cost-effectiveness, operational efficiency, and compact design [1,2,3]. A critical requirement in these applications is the accurate and adjustable control of fluid concentration, which often serves as a foundational step in experimental processes [4,5]. For example, the ability to reliably produce chemical solutions at specific concentration levels enables microfluidic systems to accelerate the optimization of sample conditions in protein crystallization, drug effectiveness assays, and immunological analyses [6,7]. This functionality has attracted broad interdisciplinary interest, motivating researchers across chemical, engineering, and biological fields to develop novel microfluidic approaches for achieving precise concentration control in fluid preparation [8,9].

Existing microfluidic platforms for fluid concentration regulation can generally be classified into three main categories [10]. The first category employs complete fluid mixing within microchannels to achieve homogeneous concentrations [11]. The second approach focuses on establishing precisely controlled concentration gradients under continuous flow conditions [12]. The third strategy involves preparing discrete target concentrations on-chip before forming customized gradient profiles off-chip [13]. Complete mixing techniques are particularly suitable for applications requiring uniform sample distribution, such as specialized material synthesis. However, their utility is limited when multiple distinct concentration levels are needed simultaneously [14,15,16]. For on-chip gradient generation, tree-shaped network architectures represent a common design solution. These systems repeatedly split and recombine incoming streams through multiple stages, creating stepped concentration variations across parallel output channels. This methodology offers notable advantages in throughput and operational stability, making it valuable for enzyme kinetic studies and protein crystallization screening. Nevertheless, the fabrication of these multi-level networks tends to be geometrically complex and manufacturing-intensive. Additionally, continuous gradient generation requires substantial sample volumes, presenting practical challenges for extended-duration experiments such as cell culture monitoring [17,18,19]. In comparison, the third approach utilizes external fields—including electric, acoustic, or hydrodynamic forces—to prepare specific concentrations on-demand, with gradients constructed off-chip from individually prepared samples. This effectively addresses the high consumption and temporal limitations of continuous gradient systems, and these active fluid and sample manipulation methods have been widely used in material synthesis, biochemistry, and so on [20,21,22]. However, these methods present their own constraints: passive hydrodynamic mixing typically requires complex channel geometries with limited operational flexibility, while acoustic techniques often depend on specialized interdigitated transducers that increase fabrication cost and complexity [23,24,25]. Consequently, there remains a clear need for alternative concentration control methods that combine precision with cost-effectiveness and operational adaptability.

The AC electroosmosis (ACEO) mechanism, which arises from the interplay between applied electric fields and induced fluid motion, has gained significant research interest for its capacity to enable flexible microscale flow manipulation [26,27,28,29,30]. This approach offers promising potential for handling diverse biochemical samples. ACEO-based devices can be readily fabricated using commercially available ITO substrates, providing an accessible platform for microfluidic applications such as cell capture, protein detection, and droplet transportation [31,32,33,34]. To address the current challenges in precise fluid concentration preparation, we introduce a novel method leveraging ACEO-driven flows for on-demand concentration control. Our microfluidic platform incorporates a simple glass–ITO electrode structure integrated with PDMS microchannels. To elucidate the underlying concentration control mechanisms, we developed a three-dimensional simulation model integrating electric, flow, and concentration fields, which enables systematic analysis of key operational parameters for fluid concentration modulation. Results demonstrate that fluid concentrations can be effectively and precisely regulated through coordinated adjustment of applied voltage and flow conditions. This ACEO-based approach thus provides a versatile platform for applications demanding flexible concentration control, such as material synthesis and protein crystallization studies.

## 2. Mathematical Formulation

### 2.1. Problem Description

**Figure 1:**
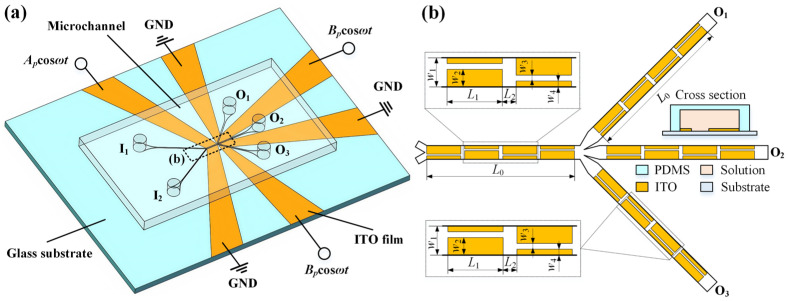
Schematic illustration of the microfluidic device for fluidic concentration control via AC electroosmotic flow. (**a**) The 3D model showing the compositions of the chip. (**b**) The key geometrical structures of the channel and electrodes, and the specific dimensions marked in (**b**) are listed in Table 1.

Figure 1a illustrates the microfluidic platform designed for continuous preparation of fluids with programmable concentrations. The device architecture integrates a PDMS microchannel bonded to a glass substrate patterned with indium tin oxide (ITO) electrodes. Critical structural dimensions and operational parameters are detailed in Figure 1b and Table 1, respectively. The electrode layout is intentionally designed with geometric asymmetry to generate non-uniform AC electroosmotic flow fields across the channel cross-section. This configuration actively enhances fluid mixing and sample transport between two co-flowing streams. During operation, two miscible fluids are introduced through independent inlets. As they progress through the main channel, their concentration profiles are progressively tuned under ACEO-mediated blending. The adjusted fluids are then bifurcated into three outlet channels, where further homogenization occurs through continued electrokinetic actuation. Through systematic regulation of both applied voltage and inlet flow rates, precise control over the final output concentrations is achieved.

### 2.2. Theoretical Background

Under the action of an alternating electric field, an electric double layer forms at the electrode surface. The tangential electric field drives the movement of the charged fluid within this double layer, which, through viscous effects, entrains the surrounding fluid, ultimately generating a three-dimensional electroosmotic flow.

*Flow field*: The fluid in the microchannel meets the following continuity and momentum equations under steady-state and laminar flow conditions:

(1) ∇⋅u=0

(2) $$-\nabla p+\mu \nabla^{2}u=0$$

where *μ* represents the apparent viscosity of the fluid, *p* denotes pressure, and ***u*** is the velocity.

In accordance with the Helmholtz–Smoluchowski formulation, the slip velocity at the electrode surface due to ACEO is expressed as [35]

(3) $$u_{slip}=-\frac{\varepsilon_{f}\tilde{\zeta }E_{t}}{\mu }$$

where *ε_f_* is the fluid permittivity, $\tilde{\zeta }$ is the zeta potential across the electric double layer, *μ* is the viscosity of the fluid, and ***E_t_*** is the tangential component of the electric field in the fluid.

Uniform fluid velocity is applied at inlet boundaries 1 and 2,

(4) $$\left\{\begin{array}{c} u_{1}=u_{1}\cdot n\\ u_{2}=u_{2}\cdot n \end{array}\right.$$

where ***n*** is the normal vector of inlet boundary.

The outlets are set to be the pressure outlet, and the other boundaries meet the non-slip boundary condition.

*Electric field*: The electric potential within the liquid domain is governed by Laplace’s equation:

(5) $$\nabla \cdot (\sigma E)=-\sigma \nabla^{2}\varphi$$

where *σ* is the fluid conductivity, ***E*** is the electric field intensity, and *φ* is the electric potential.

The potentials applied to the left and right driving electrodes are specified as:

(6) $$\left\{\begin{array}{c} \tilde{\varphi_{L}}=V_{1}\\ \tilde{\varphi_{R}}=0 \end{array}\right.$$

At the interface between the electrolyte and the electric double layer, the charging process is described by an RC circuit model, leading to the interfacial condition:

(7) $$\sigma \cdot n\nabla \tilde{\varphi }=j\omega C_{0}(\tilde{\varphi }-\tilde{\varphi_{0}})$$

where ***n*** is the unit normal vector pointing outward from the electrode surface, *ω* = 2π*f* is the angular frequency with *f* being the linear frequency, $\tilde{\varphi }$ is the potential in the bulk region adjacent to the double layer, $\tilde{\varphi_{0}}$ is the induced potential on the floating electrodes, and *C*_0_ is the capacitance per unit area of the overall electric double layer.

The capacitance per unit area of the overall electric double layer meets

(8) $$C_{0}=\frac{C_{s}C_{d}}{C_{s}+C_{d}}=\frac{C_{d}}{1+\frac{C_{d}}{C_{s}}}=\frac{C_{d}}{1+\delta }=\frac{\varepsilon_{f}}{\lambda_{d}(1+\delta )}$$

where $C_{s}$ is the equivalent capacitance of the Stern layer, $C_{d}$ is the equivalent capacitance of the Diffuse layer, δ is the capacitance ratio of the Diffuse layer to the Stern layer, and $\lambda_{d}$ is the Debye length.

The voltage drop across the electric double layer constitutes the effective zeta potential responsible for driving AC electroosmosis, expressed by

(9) $$\tilde{\zeta }=\frac{1}{1+\delta }({\tilde{\varphi }}_{0}-\tilde{\varphi })$$

All other boundaries are electrically insulated:

(10) n⋅∇φ=0

*Concentration field*: The mass transport of solute within the fluid follows Fick’s second law:

(11) $$u\cdot \nabla c-D\nabla^{2}c=0$$

where *c* is the sample concentration and *D* is the diffusion coefficient of the sample.

The mixing performance is quantified using the following expression [36,37,38]:

(12) $$M_{e}=(1-\frac{\iint_{s}\left|c_{0}-c_{\infty }\right|dA}{\iint_{s}\left|c_{\infty }\right|dA})\times 100\%$$

where *c* refers to the concentration at a specified distance from the microchannel inlet and *c*_∞_ is the concentration under fully mixed conditions. The value of Me ranges from 0 to 1, indicating a transition from a completely unmixed state (0) to a fully mixed state (1).

### 2.3. Solution Methodology

**Figure 2:**
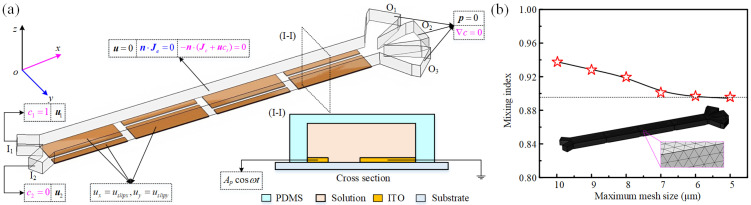
The boundary conditions and grid dependence test of the simulation model. (**a**) Setup of the boundary conditions. (**b**) The effect of mesh size on the simulation results. Simulations within the software are performed using a tetrahedral computational grid.

A three-dimensional simulation model integrating electrical, thermal, fluidic, and mass transport phenomena was implemented in COMSOL Multiphysics 6.0, a commercial finite element analysis platform. This computational model was developed to investigate the fundamental mechanisms underlying ACEO-driven microfluidic concentration control. As shown in Figure 1b, the electrode arrays in both the main and branch channels share identical geometrical configurations to ensure consistent electrokinetic mixing performance throughout the device. This symmetrical design approach guarantees that complete mixing achieved in the main channel under specific voltage and flow rate conditions can be replicated in branch channels with equivalent electrical parameters. To optimize computational efficiency while maintaining physical relevance, the simulation domain is strategically reduced to focus exclusively on the main channel section (Figure 2a). The boundary conditions governing each physical field are comprehensively specified in Figure 2a. Electrically, sinusoidal AC signals drive the electrode terminals while all channel walls satisfy electrical insulation constraints. For fluid dynamics, specified velocity profiles define the inlet boundaries, no-slip conditions apply at channel walls, and zero static pressure is maintained at the outlet. The fluid density in the numerical model is 1000 kg/m^3^, the fluid viscosity changes from 1 mPa·s to 8 mPa·s, the diffusion coefficient *D* is 2 × 10^−10^ m^2^/s, and the fluid conductivity changes from 0.001 S/m to 0.05 S/m.

To ensure computational accuracy, a systematic mesh independence study was conducted prior to numerical analysis. The three-dimensional model was discretized using tetrahedral elements, with mesh distribution automatically adapted to physical field variations within COMSOL Multiphysics 6.0. Mesh sensitivity was quantified through the mixing index (Me), which served as the convergence criterion (Figure 2b). Results indicated that simulation outcomes exhibited significant mesh dependence until element sizes reached 6 μm. Further refinement below this threshold yielded diminishing returns in accuracy while substantially increasing computational demands. Therefore, a maximum element size of 6 μm was adopted throughout subsequent simulations to balance numerical precision with computational efficiency.

**Figure 3:**
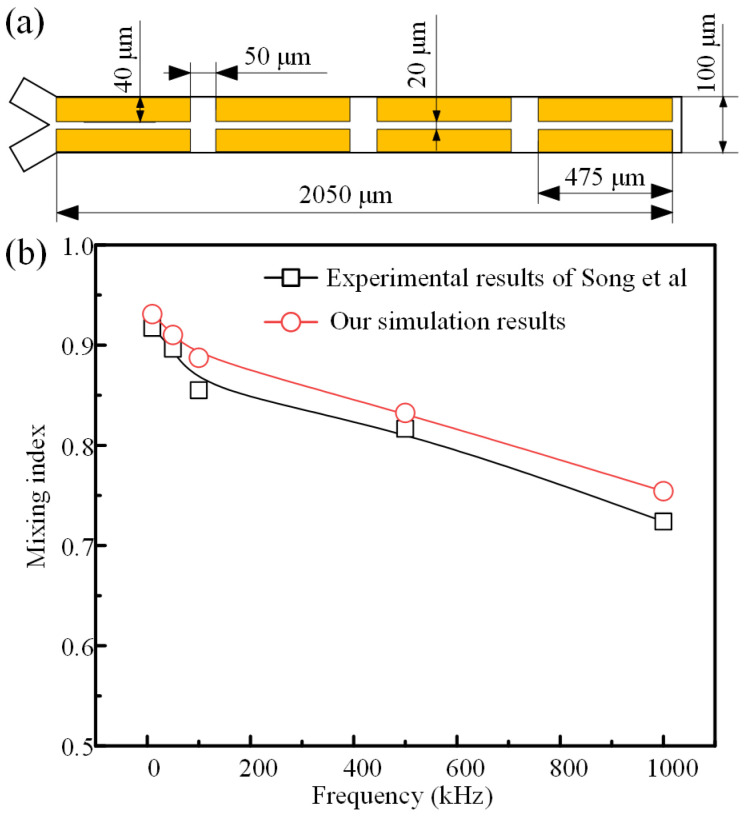
Comparison between our simulations with the experiments of Song et al. [39]. (**a**) The geometrical structure of the DC-biased AC electroosmotic mixer. (**b**) The plots of mixing index versus signal frequency (25 Vpp, 2 VDC).

To validate the accuracy of our simulations, we tried to compare our simulations with the reported experiments. Specifically, Song et al. [39] reported a DC-biased AC electroosmotic mixer, as shown in Figure 3a. We used a similar modeling method in this study to establish a simulation model. The simulation parameters were kept consistent with those of the proposed mixer. A comparison between our simulation results and the previously reported experimental data is presented in Figure 3b. It can be observed that the results are in good agreement, with a maximum deviation of less than 4.2%, which confirms the accuracy of our modeling approach.

## 3. Results and Discussion

**Figure 4:**
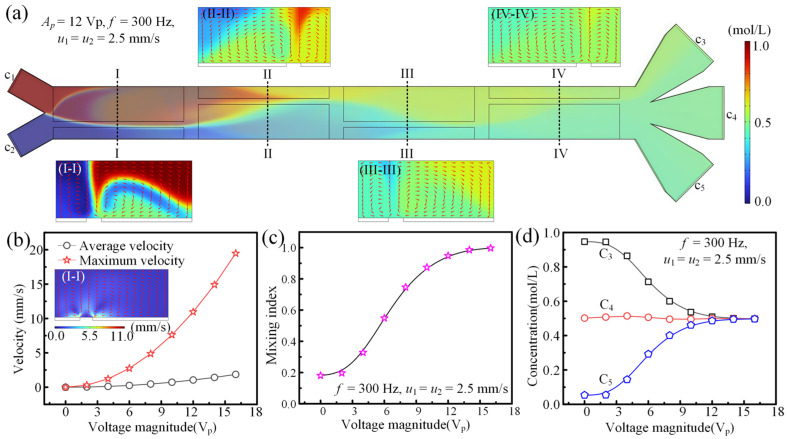
The fluid mixing and concentration modulation mechanism under AC electroosmosis. (**a**) The fluid mixing state in the main channel at *A_p_* = 12 V_p_, *f* = 300 Hz, *σ* = 0.001 S/m, and *u*_1_ = *u*_2_ = 2.5 mm/s. (**b**) The maximum and average fluid convection velocities on the I-I cross-section under different driving voltages at *f* = 300 Hz, *σ* = 0.001 S/m, and *u*_1_ = *u*_2_ = 2.5 mm/s. (**c**) The plot of the fluid mixing index at the outlet of the main channel versus driving voltage at *f* = 300 Hz, *σ* = 0.001 S/m, and *u*_1_ = *u*_2_ = 2.5 mm/s. (**d**) The concentrations of fluids entering three different branch channels under different driving voltages.

In this study, the fluid concentration in a microfluidic device is on-chip modulated via controllable AC electroosmotic flow. When the driving signal and fluid velocity are fixed at *A_p_* = 12 V_p_, *f* = 300 Hz, *σ* = 0.001 S/m, and *u*_1_ = *u*_2_ = 2.5 mm/s, the resulting fluid mixing and transport behavior is illustrated in Figure 4a. Four asymmetrical electrode pairs are arranged at the bottom of the main channel, and asymmetrical ACEO flow forms above the electrodes, which can improve fluid mixing performance by stirring fluids and disturbing fluidic interfaces. At *f* = 300 Hz and *u*_1_ = *u*_2_ = 2.5 mm/s, the maximum and average fluid convection velocities on the I-I cross-section (marked in Figure 4a) under different driving voltages are shown in Figure 4b. The intensity of ACEO flow rises with the increased driving voltage, leading to an enhanced fluid mixing index at the outlet of main channel (Figure 4c). The resultant fluid concentrations at the inlets of three branch channels are provided in Figure 4d. It can be found that the fluid concentrations are precisely regulated through voltage adjustment. This electrokinetic strategy therefore enables continuous preparation of microfluids with programmable concentration profiles, presenting a versatile platform for applications demanding precise fluidic composition control. The detailed analysis of fluid concentration modulation via ACEO is shown in the following sections.

### 3.1. The Effect of Electric Driving Signal on Fluid Concentration Modulation

**Figure 5:**
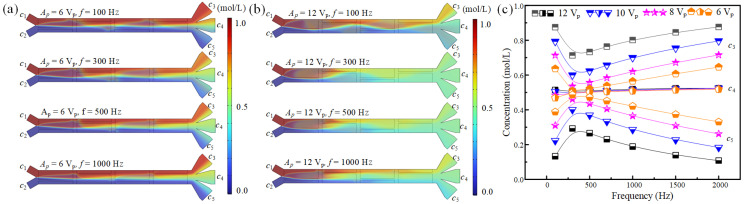
The fluid mixing and concentration modulation by voltage and frequency control. (**a**,**b**) The fluid mixing states in the main channel under different frequencies and voltages at *σ* = 0.001 S/m, *u*_1_ = *u*_2_ = 2.5 mm/s. (**c**) The concentrations of fluids entering three different branch channels under different driving voltages.

This study establishes an ACEO-based methodology for achieving programmable concentration control in microfluidic systems. Since ACEO flow-induced mixing efficiency depends on multiple interdependent parameters including axial fluid velocity, excitation voltage, and signal frequency, we performed systematic investigations to elucidate the underlying transport mechanisms. Figure 5a,b present the evolution of interfacial patterns and mass transport between two co-flowing streams under synchronized inlet velocities while varying the applied voltage magnitude and signal frequency. It can be found that the fluid stirring and mixing performance is positively affected by the increased driving voltage. This variation trend is due to the fact that the intensity of ACEO flow increases with the increased tangential electric field intensity (Equation (3)), and the improved voltage will enhance the electric field intensity in fluids.

As for the effect of electric signal frequency on fluid stirring and sample mixing performance, it is clear that their relationship is nonlinear. Specifically, the fluid mixing performance increases when the signal frequency is smaller than 300 Hz, while it begins to decrease as the signal frequency further increases (Figure 5a,b). This phenomenon is due to the fact that the velocity of ACEO exhibits a non-monotonic dependence on the driving frequency, initially increasing before decreasing. The non-monotonic variation in ACEO velocity with frequency arises from the interplay between the polarization of the electric double layer (EDL) and the AC field frequency. At low frequencies (<300 Hz), the slow field variation allows full EDL polarization but yields minimal net ion migration due to sluggish field reversal, resulting in low flow velocity. Around 300 Hz, the EDL charging–discharging synchronizes optimally with the field oscillation, maximizing the coupling between interfacial charge and tangential field to produce a peak in time-averaged velocity. At higher frequencies, the EDL can no longer follow the rapid field changes, polarization declines, and the reduced net interfacial charge causes a drop in ACEO velocity, despite the presence of the tangential field.

Figure 5c presents the average fluid concentrations in three branch channels under different driving voltages and signal frequencies, demonstrating that concentration can be effectively modulated by varying these parameters. In the studied configuration, Inlet 1 and Inlet 2 introduced solutions with concentrations of 1 mol/L and 0 mol/L, respectively. Without an applied voltage, laminar flow dominated with minimal mixing, resulting in a gradual decrease in concentration along the channel cross-section and a corresponding reduction in average concentration from *c*_3_ to *c*_5_. Upon applying an electric field, active stirring enhanced fluid mixing, causing *c*_3_ to decrease and *c*_5_ to increase with rising voltage. In contrast, *c*_4_ remained around 0.5 mol/L, largely unaffected by voltage due to its central position. Furthermore, as signal frequency nonlinearly influenced mixing performance, both *c*_3_ and *c*_5_ exhibited nonlinear variations with increasing frequency. Specifically, at an applied voltage of 12 V_p_, as the frequency increased from 100 Hz to 2000 Hz, the average concentration at *c*_3_ decreased gradually from 0.875 mol/L at 100 Hz to 0.713 mol/L at 300 Hz, and then increased again, reaching 0.876 mol/L at 2000 Hz. Therefore, the average fluid concentrations at the entrances of branch channels can be flexibly adjusted by ACEO flow.

### 3.2. The Effect of Fluid Properties on Concentration Modulation

**Figure 6:**
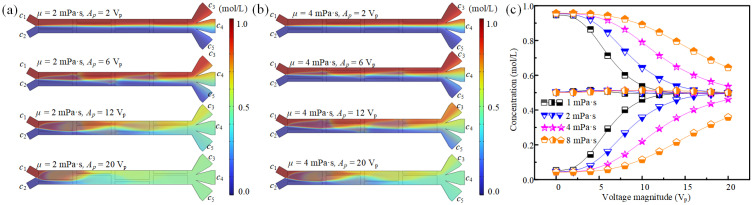
The effect of fluid viscosity on fluid concentration modulation. (**a**,**b**) The fluid mixing and sample transfer states in the main channel under different fluid viscosities and driving voltages at *u*_1_ = *u*_2_ = 2.5 mm/s, *σ* = 0.001 S/m, and *f* = 300 Hz. (**c**) The concentrations of fluids entering three different branch channels under different fluid viscosities and driving voltages.

Besides the electric signal, the intensity of AC electroosmotic flow is also greatly affected by fluid properties including fluid viscosity and conductivity. According to Equation (3), the slip velocity at the electrode surface due to ACEO is negatively affected by fluid viscosity. Thus, we further analyze the ACEO-induced fluid concentration modulation behaviors under different fluid viscosities. As shown in Figure 6a,b, at *u*_1_ = *u*_2_ = 2.5 mm/s, *f* = 300 Hz, by changing the driving voltage and fluid viscosity, the fluids in the main channel show varied mixing states. The corresponding average fluid concentrations at the entrances of three branch channels are illustrated in Figure 6c. It can be found that the average concentration *c*_4_ remains stable under different fluid viscosities and driving voltages with a value of ~0.5 mol/L. However, *c*_3_ decreases with increased driving voltage, and *c*_5_ improves with the increase in driving voltage. For instance, at *μ* = 2 mPa·s, when the driving voltage increases from 0 V_p_ to 20 V_p_, *c*_3_ declines from 0.95 mol/L to 0.5 mol/L, while *c*_5_ improves from 0.04 mol/L to 0.5 mol/L. As for the influence of fluid viscosity, it has a negative effect on fluid stirring and sample transfer, leading to the increase in *c*_3_ and the decrease in *c*_5_. For example, when the driving voltage is fixed at 10 V_p_, as the fluid viscosity rises from 1 mPa·s to 8 mPa·s, *c*_3_ improves from 0.54 mol/L to 0.89 mol/L, while *c*_5_ declines form 0.46 mol/L to 0.11 mol/L. Therefore, with the change in fluid viscosity, the concentration modulation performances under ACEO are also different.

**Figure 7:**
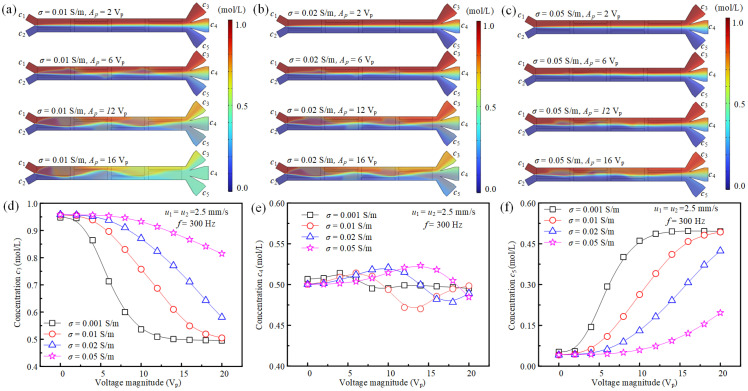
The effect of fluid conductivity on fluid concentration modulation. (**a**–**c**) The fluid mixing and sample transfer states in the main channel under different fluid conductivities and driving voltages at *u*_1_ = *u*_2_ = 2.5 mm/s and *f* = 300 Hz. (**d**–**f**) The average concentrations of fluids entering three different branch channels under different fluid conductivities and driving voltages.

As for the effect of fluid conductivity on ACEO-based concentration modulation, they are shown in Figure 7. At *u*_1_ = *u*_2_ = 2.5 mm/s, *f* = 300 Hz, it can be seen that the fluid stirring effect in the main channel decreases with the increase in fluid conductivity (Figure 7a–c). This is due to the fact that the thickness of the electric double layer decreases with the rising fluid conductivity. Subsequently, under electric field, the flow of charged fluids in the weakened electric double layer decreases, leading to a decreased fluid disturbance effect. Figure 7d–f show the average fluid concentrations *c*_3_, *c*_4_, and *c*_5_ under different driving voltages and fluid conductivities, respectively. It is clear that the fluid concentration modulation effect changes with the fluid conductivity, but *c*_3_, *c*_4_, and *c*_5_ show a different variation trend. Specifically, *c*_3_ rises with the increased fluid conductivity and decreases with the increase in driving voltage. The effect of fluid conductivity and driving voltage on *c*_4_ is very small; its value is ~0.5 mol/L under different concentrations. Whereas *c*_5_ decreases with the increased fluid conductivity and improves with the increased driving voltage. At *u*_1_ = *u*_2_ = 2.5 mm/s, *f* = 300 Hz, when the driving voltage changes from 0 to 20 V_p_, the control range of *c*_3_ changes from 0.495 mol/L~0.947 mol/L to 0.815 mol/L~0.958 mol/L as the fluid conductivity improves from 0.001 S/m to 0.05 S/m. And the concentration modulation range of *c*_5_ changes from 0.052 mol/L~0.495 mol/L to 0.041 mol/L~0.196 mol/L. Thus, the ACEO-based fluid concentration modulation effect is also greatly influenced by fluid conductivity. Regarding potential Joule heating effects under AC electric fields, these can be effectively mitigated through careful optimization of key operating parameters, such as field frequency, voltage amplitude, and solution conductivity. By doing so, excessive temperature rise can be largely avoided. And electric methods have been successfully implemented in the handling of sensitive biological samples. Therefore, the apparent Joule heat can be avoided in our fluid concentration control strategy.

### 3.3. The Effect of Fluid Velocity on Concentration Modulation

In the microchannel, the fluid stirring and mixing effect is also affected by the residence time of ACEO flow. Thus, we further study the influence of axial fluid velocity on the concentration modulation performances.

**Figure 8:**
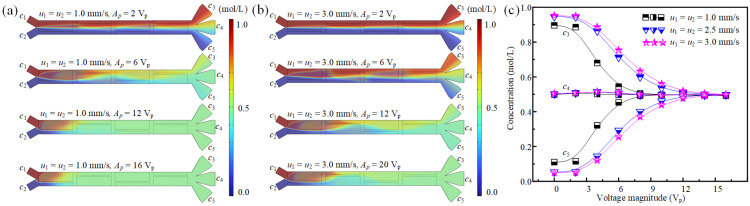
Fluid mixing and concentration modulation under varying inlet velocities and driving voltages. (**a**,**b**) Mixing characteristics and sample transfer states for two fluids at *σ* = 0.001 S/m, *f* = 300 Hz with *u*_1_ = *u*_2_, under different combinations of inlet velocity and driving voltage. (**c**) The average concentrations *c*_3_, *c*_4_, and *c*_5_ as a function of driving voltage for different *u*_1_ values.

Under the condition of synchronized and equal inlet flow velocities, the evolution of mixing and sample transport for two miscible fluids is presented in Figure 8a,b across a range of driving voltages. As the driving voltage rises, a corresponding enhancement in sample transport and mixing efficiency is observed between the two fluids. This improvement is attributed to the augmentation in the ACEO flow, which intensifies with higher driving voltages, thereby facilitating more vigorous fluid stirring and mass transfer. Conversely, an increase in the inlet velocity impedes sample transport. This occurs because a higher axial flow velocity reduces the exposure time of the fluid to the transverse ACEO flow, diminishing its effectiveness in sample transfer. Due to the stirring action of the ACEO flow, the two miscible fluids attain distinct average concentrations, designated as *c*_3_, *c*_4_, and *c*_5_, at the inlets of the three branch channels.

Figure 8c presents the average concentrations, *c*_3_, *c*_4_, and *c*_5_, measured across a range of inlet velocities and driving voltages. The data demonstrates that adjusting the driving voltage and inlet velocity provides flexible control over the average fluid concentration at the branch channel inlets. Notably, *c*_3_, *c*_4_, and *c*_5_ exhibit different relationships with voltage and axial fluid velocity. Specifically, *c*_3_ decreases with the increased driving voltage, while it improves with the increased axial fluid velocity. *c*_4_ shows a stable value of ~0.5 mol/L under different axial velocities and driving voltages. *c*_5_ rises with the improved driving voltage, while it decreases with increased axial fluid velocity. Specifically, at *σ* = 0.001 S/m, *f* = 300 Hz and *u*_1_ = *u*_2_, when the driving voltage changes from 0 to 16 V_p_, the range of *c*_3_ varies from 0.492 mol/L~0.896 mol/L to 0.499 mol/L~0.953 mol/L and *c*_5_ changes from 0.110 mol/L~0.492 mol/L to 0.048 mol/L~0.496 mol/L. This is due to the fact that the fluid mixing performance is positively affected by the increased driving voltage and negatively influenced by the improved axial fluid velocity.

**Figure 9:**
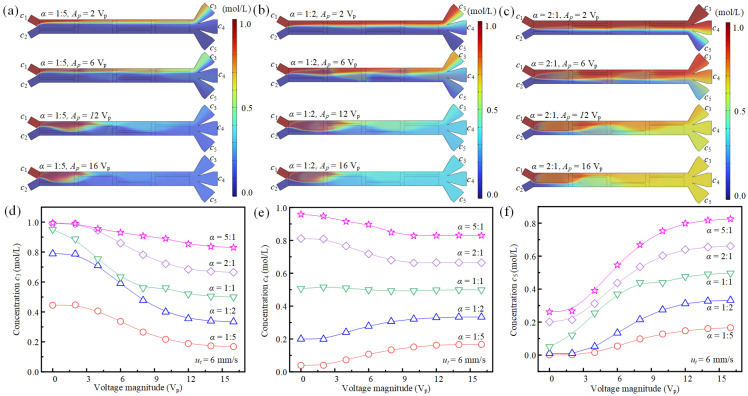
Investigation of mixing and concentration control for two miscible fluids under varying inlet velocity ratios and driving voltages. (**a**–**c**) States of fluid mixing and sample transport under different driving voltages and velocity ratios at *σ* = 0.001 S/m, *f* = 300 Hz. (**d**–**f**) Corresponding average concentrations (*c*_3_, *c*_4_, and *c*_5_) as a function of driving voltage.

Beyond configurations with identical inlet velocities, the system also allows for independent control of each inlet’s flow rate, as demonstrated in Figure 9a–c. In these experiments, with the total inlet velocity fixed at 6 mm/s, variations in the velocity ratio of *u*_1_ to *u*_2_ and the driving voltage lead to distinct sample transport patterns. These differences arise because altering the velocity ratio *α* = *u*_1_/*u*_2_ shifts the initial fluid–fluid interface and modifies the average fluid concentration within the main channel, thereby changing the starting conditions for the electrokinetic process. And the average fluid velocity in branch channels is 4 mm/s. Furthermore, Figure 9d–f reveal that the adjustable range of average concentrations at the branch channel inlets is not solely dependent on voltage but can also be tailored by the ratio of the two inlet velocities. For example, when the driving voltage improves from 0 V_p_ to 16 V_p_, as the velocity ratio α increases from 1:5 to 5:1, the tunable concentration range of *c*_3_ and *c*_4_ changes from [0.01, 0.0.08] to [0.83, 0.96], respectively, while the concentration range of *c*_5_ varies from [0.005, 0.0.08] to [0.26, 0.83]. This shift is a direct consequence of the changing proportion of the two fluids entering the system. In conclusion, the concentration at the branch channel inlets can be precisely and flexibly regulated through a synergistic control of the inlet velocity ratio and the magnitude of the applied driving voltage.

In summary, this study demonstrates that the proposed ACEO-based microfluidic platform enables flexible manipulation of fluid concentrations, thereby offering a compelling solution for applications demanding precise concentration control, such as material synthesis. Distinct from existing tree-shaped or acoustic wave-based methods, the present approach employs a straightforward channel architecture, which minimizes the risk of channel clogging. Furthermore, it affords exceptional operational flexibility through the synergistic adjustment of flow and voltage parameters. It should be noted, however, that the current configuration is subject to a limitation in throughput.

Through the above analyses, we have systematically analyzed the concentration modulation in the main channel by changing various parameters including fluid properties, electrical signals, and fluid velocity. When the fluids flow into branch channels, they can be mixed well by further energizing the electrodes in branch channels. Since the key structural features of the branch channels are the same as those of the main channel, and the average fluid velocity in the branch channels is about two-thirds of that in the main channel, therefore, a voltage sufficient to achieve mixing in the main channel is expected to be similarly effective in the branch channels.

## 4. Conclusions and Outlook

This article presents a microfluidic strategy for the on-demand and continuous formulation of Newtonian and non-Newtonian fluids with precise concentrations, leveraging ACEO phenomena. The device operates by introducing two miscible fluids—a 1 mol/L concentrate and a pure solvent—through separate inlets. The target concentration is achieved by precisely modulating the average concentration at the branch channel inlets, followed by active homogenization via ACEO vortices within the branch channels. To elucidate the underlying concentration control mechanism, a three-dimensional multiphysics model was developed, fully coupling the electric, thermal, flow, and mass transport fields.

Our analysis identifies several critical parameters governing the output concentration: fluid viscosity, inlet velocity, driving voltage and signal frequency. An increase in fluid viscosity attenuates the ACEO flow, necessitating a higher voltage range to achieve the same concentration target. Similarly, a higher inlet fluid velocity reduces the residence time for ACEO-driven mixing, also demanding an increased control voltage for equivalent adjustment. Conversely, the operable concentration modulation range can be dynamically shifted by altering the ratio of the two inlet velocities. For example, when the driving voltage improves from 0 V_p_ to 16 V_p_, as the velocity ratio α increases from 1:5 to 5:1, the tunable concentration range of *c*_3_ and *c*_4_ changes from [0.01, 0.0.08] to [0.83, 0.96], while the concentration range of *c*_5_ varies from [0.005, 0.0.08] to [0.26, 0.83].

In summary, this method enables flexible fluid preparation through the coordinated control of inlet velocities, driving voltage, and signal frequency. This technique holds significant promise for applications demanding dynamic concentration control, such as material synthesis and protein crystallization. Future work will focus on experimental validation and practical implementation to further demonstrate the feasibility and efficacy of this approach. In the future, we will systematically conduct experimental studies to demonstrate the feasibility and effectiveness of this concentration modulation method. And also the fluid mixing performances in branch channels will be systematically analyzed by experiments.

## Figures and Tables

**Table 1:** The main structural parameters of the microdevice.

Parameters	*w*_1_	*w*_2_	*w*_3_	*w*_4_	*w*_5_	*L*_0_	*L*_1_	*L*_2_
Size (μm)	180	120	20	40	40	1950	450	50

## Data Availability

The data presented in this study is available on request from the corresponding author.
